# OakRootRNADB—a consolidated RNA-seq database for coding and noncoding RNA in roots of pedunculate oak (*Quercus robur*)

**DOI:** 10.1093/database/baac097

**Published:** 2022-11-17

**Authors:** Paulina Kościelniak, Paulina Glazińska, Marcin Zadworny

**Affiliations:** Institute of Dendrology, Polish Academy of Sciences, Parkowa 5, Kórnik 62-035, Poland; Department of Plant Physiology and Biotechnology, Faculty of Biological and Veterinary Sciences, Nicolaus Copernicus University, Lwowska 1, Toruń 87-100, Poland; Institute of Dendrology, Polish Academy of Sciences, Parkowa 5, Kórnik 62-035, Poland; Faculty of Forestry and Wood Technology, Poznan University of Life Sciences, Wojska Polskiego 71a, Poznan 60-625, Poland

## Abstract

The degree to which roots elongate is determined by the expression of genes that regulate root growth in each developmental zone of a root. Most studies have, however, focused on the molecular factors that regulate primary root growth in annual plants. In contrast, the relationship between gene expression and a specific pattern of taproot development and growth in trees is poorly understood. However, the presence of a deeply located taproot, with branching lateral roots, can especially mitigate the effect of insufficient water availability in long-lived trees, such as pedunculated oak. In the present article, we integrated the ribonucleic acid (RNA) sequencing data on roots of oak trees into a single comprehensive database, named OakRootRNADB that contains information on both coding and noncoding RNAs. The sequences in the database also enclose information pertaining to transcription factors, transcriptional regulators and chromatin regulators, as well as a prediction of the cellular localization of a transcript. OakRootRNADB has a user-friendly interface and functional tools that increase access to genomic information. Integrated knowledge of molecular patterns of expression, specifically occurring within and between root zones and within root types, can elucidate the molecular mechanisms regulating taproot growth and enhanced root soil exploration.

**Database URL** https://oakrootrnadb.idpan.poznan.pl/

## Introduction

Several adaptations are required to optimize root architecture to efficiently acquire water from either deep or shallow soil layers. This is especially true for long-lived *Quercus robur* in order to overcome water deficit challenges (1). The ability to develop a root system that enables foraging for water from deep soil layers that have higher water content for much longer periods of time than shallow, subsurface layers of the soil, is crucial to the survival of some long-lived tree species (2–7). This adaptation is of particular importance given increasing frequency of insufficient precipitation and long-lasting, extending cycles of drought (5, 9). The development of long taproots that enhance water acquisition and supply it to above-ground organs requires endogenous mechanisms that regulate root growth toward deep soil layers with high water content (10, 11). Mechanisms directing root growth toward wet soil layers include (i) taproot apical zone molecular pathways driving the ability of root tips to grow deeper and (ii) a molecular network coordinating root tip growth in response to moisture deficit and growth in the elongation zone to optimize water uptake.

The ability of roots to grow deeper beginning at the early stages of tree growth is determined by molecular drivers that coordinate and mediate root growth through long-distance signaling (12). Some of these factors include genes that control plant growth at every stage of tree development, as well as factors that control the transcription and translation of these genes, such as transcription factors (TFs) and noncoding ribonucleic acids (ncRNAs), in response to a variety of molecular and environmental signals (13, 14). Although ncRNAs are nonprotein coding molecules, they play a significant role in controlling root system architecture by regulating transcription, alternative splicing, microRNA (miRNA) activity, transcript stability and the translation of messenger RNA (15). Identifying root-related coding and ncRNAs and their function is crucial to elucidating regulatory mechanisms controlling deep taproot growth, as well as root system architecture overall, which not only determines root water absorption ability but also the pattern of root growth in response to root injury.

Taproots can be lost at early as well as at late developmental stages of a root system, as a result of biotic and abiotic stresses, as well as commonly used management procedures in container nurseries that injure taproots. Oaks deprived of taproots are more susceptible to water deficit, as they have a decreased ability to uptake water from deep soil layers (4, 5). The ability of containerized seedlings to foster the growth of fine roots to deeper soil layers and reconstitute taproot growth in a significant fraction of seedlings (5, 16, 17) suggests the capacity to restore taproot growth. Determining the factors that regulate taproot growth and enable regrowth of a taproot in container seedlings after they are planted in the field can provide a mechanistic understanding of the effect of nursery management practices on seedling growth and its potential impact on subsequently managed forest stands.

Despite the relatively good understanding of the processes regulating root growth in short-living annual plants, the same level of understanding of these processes in long-lived woody plants that have to face numerous episodes of drought during their lifespan and depend more heavily on deeply located water sources remains to be investigated or explored. Understanding the groups of genes and the factors regulating their expression used by oak trees to maintain taproot growth for many years requires the identification of root zone-specific gene expression that enables the maintenance and cessation of root growth when needed. The ability to access whole records of the global expression of coding sequence (CDS) and non-CDS expressed during root growth, rather than just bits of information, would help to identify and assess the role of signaling network–mediated taproot growth strategies, including the coordination that exists between root tip sensing and root elongation. This ability now exists due to the development of high-throughput sequencing technologies, such as next-generation sequencing, and the deposition of the acquired sequence data in publicly available databases, especially that a whole genome sequence for *Q. robur* is currently available on the Quercus Portal platform (https://quercusportal.pierroton.inra.fr/). This allows for more accurate transcript assembly and expression testing.

Existing databases for plant species typically contain information for only one type of RNA, coding or noncoding (12, 18). The QuercusMap database contains single tree genotypic and phenotypic data of offspring belonging to oak mapping pedigrees. The genotypic data comprise various markers (Amplified Fragment Length Polymorphism, Random Amplified Polymorphic DNA, Single - sequence repeats, Single Nucleotide Polymorphisms and Polymerase Chain Reaction - Restriction Fragment Length Polymorphism) traditionally used for mapping purposes (http://mapedigree.pierroton.inra.fr/qmap/). Another oak database CMAP contains genetic map data on oak pedigrees, and Quantitative Trait Loci positions for a variety of traits assessed in field tests (https://arachne.pierroton.inra.fr/cgi-bin/cmap), with limited specific information about genes or a group of genes regulating the traits. Another database expressed sequence tag (EST) contains files for three sets of unigenes constructed for the *Quercus* data related to oak pedigrees. This database also contains three versions (called Oakcontig). Version 1 (OCV1) (http://genotoul-contigbrowser.toulouse.inra.fr) contains a comprehensive collection of ESTs from oaks (19). The advanced version (OCV2) is a reference library that catalogs differential gene expression in *Q. robur* during controlled biotic interactions and is primarily used for quantitative transcriptomic profiling of oak roots in ectomycorrhizal symbiosis (20). OCV3 contains a *de novo* assembly of the oak transcriptome, focused on the molecular mechanisms involved in dormancy release in buds (21). The TreePop database contains passport, phenotypic and genotypic data of individual oaks within a single population, Instensive Study Plot or Instensive Study Sites (https://treepop.pierroton.inra.fr/). Unfortunately, there is a limited opportunity when using this database to determine the genes involved in the development of oak roots. Another database Oak provenance contains passport data and phenotypic assessments of *Quercus petraea* and *Q. robur* provenances and provenance tests established in Europe (https://oakprovenances.pierroton.inrae.fr/). Another available reference is the GD^2^ database, which contains genetic and georeferenced passport data of different genetic units in natural populations (http://gd2.pierroton.inra.fr/). This is a georeferenced database, however, less convenient as a good gene identification tool. The SSR database containing deoxyribonucleic acid (DNA) sequences of microsatellite motifs and primer DNA sequences, as well as information on candidate genes and their single nucleotide polymorphisms, is very useful (http://ssrdatabase.pierroton.inra.fr/home). One of the most interesting databases is CorkOakDB released in 2018 by the GENOSUBER consortium that contains the first draft genome of *Quercus suber* and allows genome browsing and gene searches (22). The portal (https://corkoakdb.org/) provides the ability to search and explore curated genomic and transcriptomic data on *Q. suber* but only has limited use for obtaining information pertaining to primary root growth (23). Raw data deposited in publicly accessible databases often require specialized data processing software, as well as programming skills and computers with high processing power, which can significantly limit their utility. These limitations highlight the need to create databases containing data that are available to the user in a simple, direct and easy-to-understand manner. Therefore, we endeavored to create a database and user-friendly interface that provides the ability to conduct a detailed analysis of the available data, as well as a comprehensive examination of genes, transcripts, proteins and miRNAs both separately and in the context of an interrelated network.

The objective of the *Q. robur* oak root database (OakRootRNADB) was to integrate RNA sequencing (RNA-seq) data for genes, transcript RNA sequences encoding oak proteins, and long ncRNA (lncRNA) and miRNAs (based on pre-miRNAs). In addition to protein-encoding transcripts, OakRootRNADB contains information about known and new miRNAs, as well as lncRNAs. It provides a user-friendly interface that allows one to browse transcripts, genes and miRNAs involved in oak root growth. In addition, the identification of TFs, transcriptional regulators (TRs) and chromatin regulators (CRs), as well as the predicted cellular localization of transcripts, is also available. OakRootRNADB provides the opportunity to broaden our knowledge about the root system of trees and to assess the potential role of genes and non-CDSs in mechanisms that mediate growth and a variety of other processes during taproot and lateral root development. The portal for the database can be accessed at http://oakrootrnadb.idpan.poznan.pl.

## Material and methods

### Plant material and sample collection

Two-month-old seedlings of *Q. robur* were collected and used for RNA-seq. Plants were grown in a large, semi-closed, foil greenhouse located at the Arboretum of the Institute of Dendrology of the Polish Academy of Sciences in Kórnik, Poland. Roots were grown in a clear-walled rhizotron chamber (30 × 50 cm), filled with a growing medium of peat and perlite (proportions 5:1 volumetric proportion), deacidified with dolomite and enriched with 2.5 kg/m^3^ slow-release fertilizer (Osmocote 15-9-12-2 N-P-K-Mg, with trace nutrients). The rhizotrons were constructed using two transparent plexiglass plates held 2–3 cm apart by thick-walled plastic tubing to provide adequate space for the growing roots. Waterlogging was prevented by providing drainage in the bottom of each rhizotron. The rhizotrons allowed us to record root growth measurements in the same seedlings over time without disturbing the root system. Additionally, one acorn was sown per container (180 mm high, 5 mm wide, 0.275 dm^3^) in the same growing medium under similar growth conditions that were used for the acorns that were sown in the rhizotrons. The containerized seedlings were subjected to root growth inhibition by air pruning. In the spring of the following year, 1-year-old containerized seedlings were transplanted to a rhizotron (one per rhizotron), without cutting the roots to monitor factors involved in the regrowth of a taproot in container seedlings. These samples were designated as transplanted seedlings.

Seven- and eight-week-old seedlings growing in each system (rhizotron or container) were harvested in three replicate time points (early spring and summer in 2019). Each taproot was classified at its time of harvest based on its length: short (5–9 cm), medium (9.5–15 cm) and long (>15.5 cm), as well as on its morphology: normal or thick (taproots that were thicker than normal taproots and still actively growing), and vitality: active or dying (typically container taproots that had reached the bottom of the container). The same root classification approach was used for oak seedlings that had been initially grown in a container and were then transplanted to a rhizotron in 2020. In the case of transplanted seedlings, roots were harvested 8 weeks after transplantation.

The root system of harvested seedlings was gently washed with deionized, autoclaved water (ddH_2_O) to remove adhering soil, and then the meristematic and elongation zones of taproots and lateral roots were separated and immediately frozen in liquid nitrogen and stored at −80°C until RNA extraction.

The samples were combined into two sets. The material in Dataset 1 (Table 1) was from the meristematic zone of all classes *Q. robur* taproots and lateral roots of seedlings grown in a container or a rhizotron system in 2019. The samples were labeled: 1K_kr_NN_kon_1 according to the formula, Harvest date → Type of root → Root length → Root vitality → Type of cultivation → Biological replicates as detailed in Table 1.

**Table 1.** Classification of samples in Dataset 1

Harvest date	Type of root	Root length	Root morphology/vitality	Type of cultivation
1/2/3	K/KB	kr/sr/dl	NN/NT/TN/TT	kon/rh
1—first harvest date: 7 weeks old plant (sown in early spring)	K—meristematic zone of the taproot	kr—short root (5–9 cm)	NN—normal roots	kon—growing in containers
2—second harvest date: 8 weeks old plant (sown in early spring)	KB—lateral root	sr—medium root (9.5–15 cm)	NT—dying root	rh—growing in rhizotron
3—third harvest date: 8 weeks old plant (sown in early summer)		dl—long root (>15.5 cm)	TN—thick root	
			TT—thick and dying root	

The material in Dataset 2 (Table 2) was from the meristematic and elongation zones of taproots and lateral roots of *Q. robur* growing in either a container or a rhizotron system as the acorns grown in 2019 (with the exception that 2-year-old acorns were sown, derived from the same lot of acorns that had been used in the previous year), and oak seedlings that had been initially grown in a container in 2019 and then transferred to a rhizotron in 2020. The samples were labeled: K_kr_NN_kon1 according to the formula, Type of root → Root length → Type of cultivation → Biological replicates as detailed in Table 2.

**Table 2.** Classification of samples in Dataset 2

Type of root	Root length	Root morphology/vitality	Type of cultivation
K/KB/SW	kr/sr/dl	NN/NT/TN/TT	kon/rh/krh
K—meristematic zone of the taproot	kr—short root (5–9 cm)	NN—normal condition roots	kon—growing in containers
SW—elongation zone of taproot	sr—medium root (9.5–15 cm)	NT—dying root	rh—growing in rhizotron
KB—lateral root	dl—long root (>15.5 cm)	TN—thick root	krh—growing in a container and then transplanted to a rhizotron
		TT—thick and dying root	

### RNA isolation, library preparation and RNA-seq

**Figure 1.**
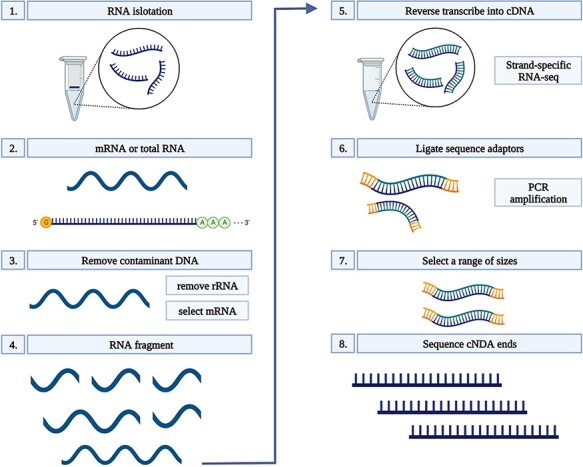
RNA-seq workflow. 1) Isolate the mRNA or total RNA from root tissue; 2) Quality and quantity control of mRNA or total RNA; 3) Eliminate DNA contamination from RNA samples using DNase; 4) Choose an appropriate kit for library preparation based on the type of RNA. For mRNA with poly-A tail, a mRNA purification kit was used to isolate mRNA with a poly-A tail; 5) Random fragment purified RNA for short read sequencing; 6) Reverse transcribe fragmented RNA into cDNA; 7) Ligate adapters onto both ends of the cDNA fragments. 8) PCR amplification of cDNA and selection of fragment sizes between 200-400 bp. Conduct paired-end sequencing of the cDNA libraries (Martin and Wang 2011).

Total RNA was extracted from 100 mg root tissue of each sample using Ribospin (GeneAll Biotechnology, Seoul, South Korea), with a DNAse treatment, according to the manufacturer’s instructions. RNA quality was assessed on 1% agarose gels. The quality and quantity of RNA were further verified prior to library constructions using a NanoDrop 1000 spectrophotometer (Thermo Scientific, Wilmington, DE, USA), and an RNA integrity number (RIN) was determined using a 2100 Bioanalyzer (Agilent, Santa Clara, CA, USA) and a small RNA kit (Agilent Santa Clara, CA, USA). Only RNA samples with a RIN of  ≥8.5 were used for complementary DNA (cDNA) conversion. Each cDNA library was constructed using a TruSeq Stranded mRNA LT Sample Prep Kit and sequenced on a NovaSeq platform (Illumina, San Diego, CA, USA) in the 150-bp PE mode. Sequencing was conducted by Macrogen (South Korea). All libraries were constructed using three biological replicates which resulted in a total number of 75 libraries in Dataset 1 and 90 in Dataset 2 (Figure 1) (24).

### Quality control and preprocessing of raw sequence data

Quality reports were generated with FastQC v0.11.5. The reads were then subjected to quality filtering and adapter trimming using BBDUK 2 v38.41 with the following settings: qtrim = w, trimq = 20, maq = 10, *k* = 23, mink = 11, hdist = 1, tbo, tpe, minlength = 50, removeifeitherbad = t. This was followed by removal of reads that mapped to *A. thaliana* ribosomal RNAs using Bowtie 2 v2.3.5.1.

### RNA-seq: *ab initio* transcriptome assembly and estimation of transcript expression

Reads were mapped against the *Q. robur* genome Qrob_PM1N.fa using Spliced Transcripts Alignment to a Reference (STAR) 2.5.3a using the settings described in the STAR manual (https://github.com/alexdobin/STAR) (25). The resulting BAM files, one per sample, were then subjected to *ab initio* transcriptome assembly using StringTie v1.3.3b, observing the reads strandedness. The resulting The Gene transfer format (GTF) files, one per sample, were then merged using StringTie into a single oak transcriptome in a GTF format. RNAs with no genomic strand assigned were discarded. Each transcript was given an ID of the type MSTRG.1.1. This designation indicates that it is a transcript of a MSTRG.1 gene. The first number is the gene identifier, and the second number is the identifier for the specific transcript that had been assigned to that gene. Expression values were obtained using RNA-Seq by Expectation- Maximization with Bowtie 2 as a mapper and observing the reads strandedness. The resulting files refer to expression on the gene level and the transcript level. The expression values are shown in three ways: expected count, the number of paired reads mapped to a given gene/transcript; transcripts per million (TPM), the number of paired reads mapped to a gene/transcript and normalized to the size of sequenced data and transcript/gene length; and fragments per kilobase of exon per million fragments mapped, the number of paired reads mapped to a gene/transcript and normalized to the size of sequenced data and transcript/gene length (a different formula is used there as compared to the case of TPM values).

### RNA-seq: transcript annotation

Annotation of the assembled transcripts was performed with Trinotate v 3.0.2. In the first step, the transcript sequence was searched against Swiss-Prot proteins, a nonredundant and manually curated set of proteins in the UniProt database. BLASTX in the Basic Local Alignment Search Tool (BLAST) package was utilized, with -max_target_seqs 1 option. Open reading frames (ORFs) were predicted with TransDecoder v 5.0.1, and the predicted protein sequences were searched against Swiss-Prot using BLASTP in the BLAST package (-max_target_seqs 1 option was used). Next, the transcripts were searched against protein domains in the PFAM database using hmmscan with default settings. Trinotate v 3.0.2 was also used to annotate the transcripts using the Kyoto Encyclopedia of Genes and Genomes (KEGG) database. The results obtained from BLAST and PFAM were used to further annotate the transcripts using the Gene Ontology (GO) database.

### Identification of ncRNAs

Transcript sequences in FASTA format were extracted from the oak genome, based on the GTF file data of the assembled oak transcriptome. lncRNA identification was performed using the following settings as implemented in in-house Python scripts (26, 27). Transcripts shorter than 200 bases were first removed. Next, transcripts containing ORFs as identified using TransDecoder v5.0.2 with -m 100 (minimum protein length; default: 100) and -S (strand-specific) options were discarded. Transcripts classified as coding by coding potential calculator (version 0.9-r2) with default settings were also discarded.

Pre-miRNA sequences were downloaded from miRBase 22, and Us in the sequences were converted to Ts. A BLASTN search conducted, using the transcriptome as a database and the downloaded pre-miRNAs as a query. An *E*-value threshold of 1e-5 was set, and an “-m 8” parameter was used to obtain a tab-delimited output file. The output file was then parsed and filtered with an in-house Python script. Hits were required to be in a sense orientation. An *E*-value of <1e-8 was also required (manual inspection of the results indicated that a threshold of 1e-5 was too liberal, as a substantial number of hits against animals were obtained). For each transcript, only the best hits, in terms of *E*-value, were kept. Finally, to filter hits against animal pre-miRNAs, which were relatively scarce, only hits against plants were retained (Viridiplantae). This was accomplished using the organismstxt file downloaded from miRBase that links species code (like ath for *Arabidopsis thaliana*) to species names and clades.

### Identification of TFs, TRs and CRs

Identification of TFs, TRs and CRs was accomplished using PlantTFcat with default settings. The prediction was done for protein sequences predicted with TransDecoder. The resulting list is available in the database annotation directory and contains the following fields: (i) Family, such as C2H2, WD40-like and SET; (ii) Family_type, such as TF and chromatin remodeling; (iii) Sequence_Acc: ID of input protein sequence; (iv) Domains, such as IPR001214 (protein domain IDs from InterPro database) and (v) Sequence_Annotation: an extended name for the input protein sequence.

### Prediction of cellular localization of the transcripts

mRNALoc was used for predicting the cellular localization of transcripts. Default settings were used with the exception of threshold for “Prediction score”, which was set to 0.0. Lower thresholds result in lower specificity of the predictions, but higher thresholds did not produce any output predictions for a number of RNAs. The data are grouped into predicted localization categories (cytoplasm, nucleus, endoplasmic reticulum, extracellular or mitochondria); however, if the prediction score is below the threshold (<0.0), a “No Localization Found” designation is provided.

### Validation of gene expression by reverse transcription-quantitative polymerase chain reaction

First-strand cDNAs were synthesized using 1 μg of total RNAs as a template and SuperScript III Reverse Transcriptase (Invitrogen, Carlsbad, CA, USA). Total RNA and oligo d(T) primers were then subjected to heat at 65°C for 5 min, incubated at 50°C for 60 min and extension at 70°C for 15 min. Synthesized cDNA samples were diluted five times prior to the reverse transcription-quantitative polymerase chain reaction (RT-qPCR). RT-qPCR analysis was used to validate the RNA-seq results on gene expression.

The RT-qPCR was conducted using a SensiFAST Probe No-ROX Kit (Bioline, UK) following the manufacturer’s protocol. The volume of the reaction mixture was 11 μl, consisting of 5.2 μl of 2x SensiFAST qPCR Master Mix, 0.02 μM of each primer, 0.1 μM of a specific Universal Probe Library (UPL) probe and 5 μl of diluted cDNA. The RT-qPCR analysis was carried out on a LightCycler480 (Roche, Switzerland) under the following conditions: 95°C for 10 min, 45 cycles of 95°C for 10 s, 58°C for 30 s and 72°C for 1 s. Two biological and three technical replicates were used for each gene in each sample group. *Ubiquitin* and *elongation factor* were used as reference genes for normalization. Primers and UPL probes used in the RT-qPCR analysis were designed using the Universal Probe Library Assay Design Center (Roche, Switzerland) and are listed in Supplementary Table S1. Expression levels in the RT-qPCR analyses were determined using LightCycler480 software (Roche, Switzerland).

### Database implementation and testing

The OakRootRNADB interactive database was constructed using the relational database management system MariaDB (https://mariadb.org/), PHP 7.4. (https://www.php.net/), Bootstrap 4 framework (https://getbootstrap.com/), HTML 5, CSS 3, JavaScript and jQuery 3.6.0 (https://jquery.com/). The interactive JavaScript Chart.js library (https://www.chartjs.org/) was used for visualization of the expression data. National Center for Biotechnology Information (NCBI) BLAST+ 2.8.1 was employed as a local alignment search tool (28, 29). The database was tested, and it was determined that it could be successfully run on several different web browsers, including Google Chrome, Mozilla Firefox, Microsoft Edge, Vivaldi and Opera. The responsive web design facilitates the use of the database on mobile devices (https://www.ncbi.nlm.nih.gov/books/NBK131777/).

## Results

### Utilization of the database

#### Data sources and generation in OakRootRNADB

OakRootRNADB was created based on transcriptomes of pedunculate oak roots. Two different zones of the taproots (meristematic and elongation) and lateral roots were sampled. Taproots were classified according to their length (short 5–9 cm, medium 9.5–15 cm and long >15.5 cm) to determine the molecular networks associated with root elongation. The experimental design was designed (i) to determine the global changes in gene expression at the postemergence steps of root development, (ii) provide information on the potential regulation of taproot and lateral root growth, as well as similarities/differences in molecular mechanisms regulating growth in both root types, and (iii) identify the genes being expressed during root establishment at different time points. Three independent samples (biological replicates) of taproots and lateral roots were collected separately in early spring and summer to determine if sowing date had an impact on root development, and how the postemergent stage of growth (i.e. roots of different lengths) affects the profile of gene expression. We also took into account root morphology, as we observed that root elongation was inhibited in some of the roots growing in the container system but continued to increase in thickness. We assume that ceasing growth before reaching the bottom of container would prevent them from dying, which occurs as a result of air pruning. The experimental design allowed us to determine factors responsible for the cessation of growth, and factors determining the restoration of taproot growth after air pruning occurred in container-grown seedlings which were subsequently planted in the field. In addition, our approach also provided information pertaining to the molecular processes involved in root dieback.

After sequencing and preliminary data analysis, data corresponding to coding RNAs and ncRNAs were deposited as raw sequences in the NCBI Gene Expression Omnibus (GEO) database, after which analysis-ready data were uploaded to the OakRootRNADB database. Supplementary Table S2 lists details of the data deposited in NCBI’s GEO and the OakRootRNADB.

The final transcriptomes obtained after sequencing, filtering, assembly and analysis comprised 145 538 transcripts belonging to 35 397 genes. Additionally, 24 593 lncRNAs and 225 miRNAs (based on pre-miRNA) were identified in the dataset. This level of ncRNAs is expected for higher organisms, such as plants. The transcripts were evenly mapped to the reference genome. Transcript assembly was accomplished using the *ab initio* (reference-based) method. The statistics on the transcriptome assembly are presented in Table 3. Over 900 000 transcripts and proteins, including over 16 000 unique transcripts, were annotated to PFAM, KEGG and GO databases using BLASTX and BLASTP (Table 3).

**Table 3.** Summary of protein-coding transcripts deposited to date in OakRootRNADB and annotated using various open access databases

Public database	Number of annotated Differential Gene Expression (DEGs)
BLASTP	60 769
BLASTX	70 861
GO_BLAST	8490
GO_PFAM	305 261
KEGG	56 153
PFAM	150 302

**Figure 2.**
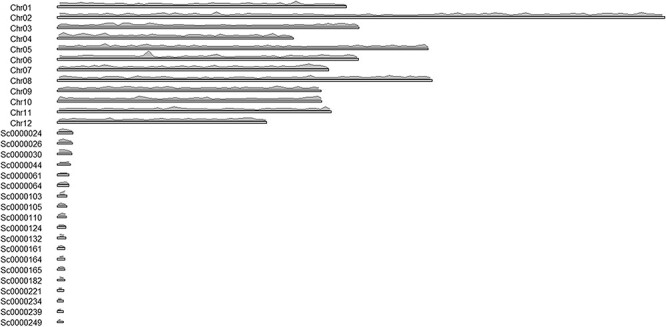
Distribution of transcripts on chromosomes.

The advantage of the *ab initio* approach is that it provides information pertaining to the localization of the transcripts, on paralogs and splicing forms, and exon-intron structure of genes. The downside of this approach is that some genes may be missing due to an incomplete genome. The transcript density on chromosomes was visualized using the karyoploteR library in the R environment (Figure 2). The shortest contours were omitted in the illustration to improve its clarity.

A total of 5349 TFs were identified in the dataset, belonging to major TF families, such as C2H2, WD40-like, MYB-HB-like, AP2-EREBP, bHLH and others (Table 4). A total of 39 of the TFs have been identified as transcription regulators, belonging to the SAP and Homeodomain-LIKE families, and 316 of the TFs have been classified as CRs. The identification of the cellular localization indicated that the identified transcripts were potentially localized to the cytoplasm, nucleus, endoplasmic reticulum, extracellular and mitochondria.

**Table 4.** Transcripts identified as TFs

Family type	Number
C2H2	900
WD40-like	647
MYB-HB-like	328
AP2-EREBP	187
bHLH	164
C3H	149
Hap3/NF-YB	129
bZIP	126
GRAS	95
NAM	95
WRKY	90
Homobox-WOX	82
FAR	72
B3-Domain	53
HSF-type-DNA-binding	52
MYB/SANT	46
C2C2-CO-like	36
C2C2-Dof	33
AS2-LOB	31
MADS-MIKC	30
TCP	30
TIFY	29
SBP	28
MYB	27
ARF	26
C2C2-GATA	26
GRF	22
TUBBY	22
Homeodomain-TALE-KNOX	19
Homeodomain-TALE-BEL	18
GARP-G2-like	16
HD-ZIP	15
GAGA-Binding-like	13
Nin-like	13
BES/BZR	12
ZF-HD	12
CG1-CAMTA	11
Hap2/NF-YA	11
GeBP	10
E2F-DP	9
Znf-LSD	9
STY-LRP1	8
MADS-type1	6
RAV	6
ssDNA-binding-TF	6
C2C2-YABBY	4
EIL	4
S1Fa-like	2
MYB-related	1
STAT	1

#### Database organization

The OakRootRNADB database was designed with a user-friendly interface that allows one to view data directly in the database and download the data from the website in various formats. On the main page, there is a quick search (Search) of the deposited sequences (transcript, gene or miRNA). Below that, there is a description of the database which includes a description of the data contained in it (Datasets 1 and 2) and relevant information pertaining the project (Project). Information on all persons involved in the creation of OakRootRNADB (People), as well as project financing (Funding) and contact information for the corresponding author (Contact), is also provided. Accessing the main components of the database is possible using tabs located at the top of the main page. The tabs are labeled: Transcript, Gene, miRNA, BLAST and Download (Supplementary Figure S1).

**Figure 3.**
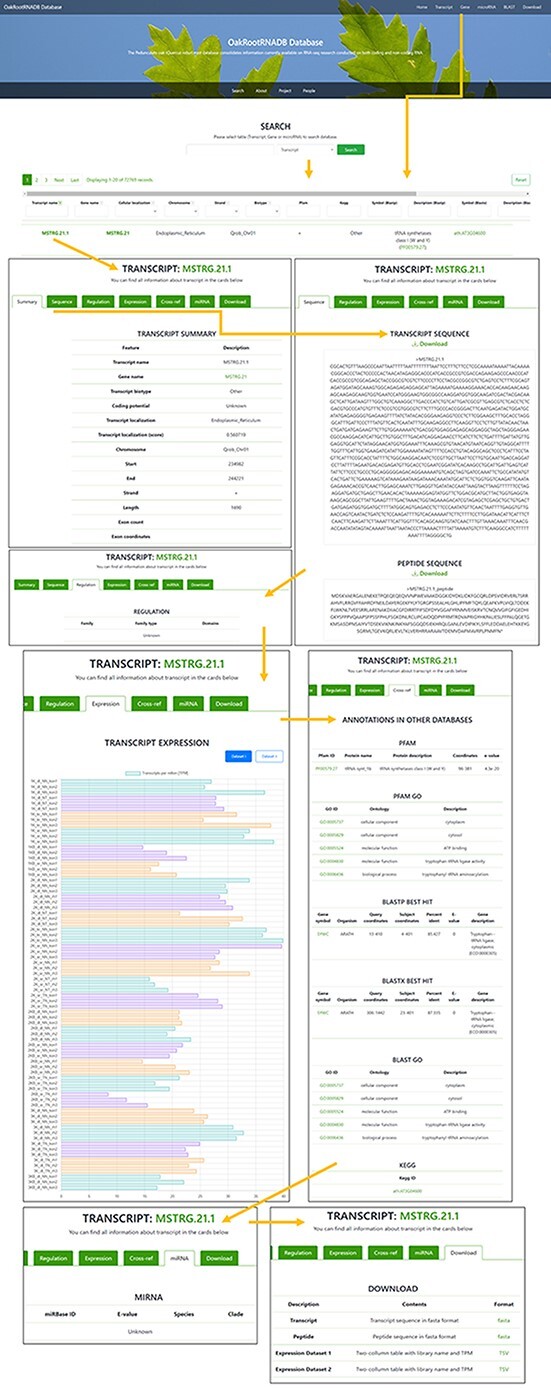
Screenshot of the information provided on the Transcript page of the OakRootRNADB.

A total of 72 769 records have been deposited in the transcript section that can be filtered by gene name, cellular localization, chromosome localization, strand, biotype (other or lncRNA), peptide, regulation or miRNA (if found). The Gene tab can be accessed directly from the Gene page through a hyperlink provided in the name of the gene. There is also a filter for annotation derived from the following databases: PFAM and KEGG (including the hierarchy), BLASTP and BLASTX (Symbol and Description). The Transcript name also includes a hyperlink to a page with more information about the transcript. In the Transcript tab, you can find more information about the transcript (Summary), the sequence of the transcript and the protein it encodes (if available), along with the ability to download the data in FASTA format. The Regulation tab provides the gene family to which the transcript belongs (if found) with a reference. The Expression tab provides information on the expression of the transcript in each of the sequenced libraries used to generate the database along with a legend. The data on transcript expression can also be downloaded in the form of a table. The Cross-ref tab provides annotation information from databases such as PFAM, PFAM GO, BLASTP, BLASTX, BLAST GO and KEGG, along with hyperlinks to each database. If the transcript was a ncRNA transcript, the miRNA tab provides information on the miRNA (if found) with a hyperlink to the miRBase database. The Download tabs provide the ability to download the nucleotide or amino acid sequence in FASTA format and information on the expression level in the libraries used to generate the database (Figure 3).

In the Gene section, as in the Transcript section, the data can be filtered by chromosome, start and end, and strand. The Gene section contains 35 397 records, corresponding to the number of identified genes. Genes are also annotated to the following databases: BLASTP, BLASTX and PFAM. The gene name also contains a hyperlink to a page with more information about the gene (Summary) along with a hyperlink to the transcript. The Sequence tab provides the sequence of a gene and the protein it encodes in FASTA format, which can be downloaded. The Expression tab displays information on the level of gene expression in each of the libraries used to construct the database, along with the legend. The data on gene expression can also be retrieved as a table. The Download tab allows one to quickly download the nucleotide sequence of all the gene transcripts or the amino acid sequence for the proteins encoded by the transcripts in a FASTA format, as well as the expression level of the deposited transcripts (Supplementary Figure S2).

Two hundred twenty-five records were deposited in the miRNA section, based on the number of miRNAs that were identified (based on pre-miRNA). The list of identified miRNAs can be searched by sequence identifier (miRNA), RNA sequence (Transcript name and Gene name), *E*-value, Species and Clade. A hyperlink is provided in the miRNA identifier to the miRBase database where detailed information about a given miRNA in other organisms can be found. This part of the database is the least comprehensive due to the limited data obtained in our miRNA analysis (Supplementary Figure S3).

**Figure 4.**
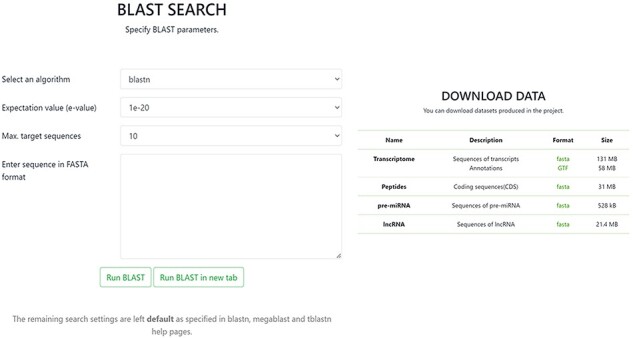
Screenshot of BLAST and Download pages in the OakRootRNADB.

In the BLAST section, it is possible to conduct a BLAST query by sequence with the option to set specific BLAST parameters. The Download tab allows one to download in FASTA format any of the data contained in the database, including sequences of transcripts with annotations, CDSs of peptides, sequences of pre-miRNA and sequences of lncRNA (Figure 4).

In addition to its user-friendly interface, the OakRootRNADB database provides the ability to conduct a data analysis through the website directly or by downloading the data from the database. Hyperlinks to other annotated databases enable a sequence to be quickly identified. Each transition to other pages in the database or other databases is simply done by opening a new tab, which prevents readers from losing the visible information already obtained.

#### Validation of the RNA-seq results deposited in OakRootRNADB

**Figure 5.**
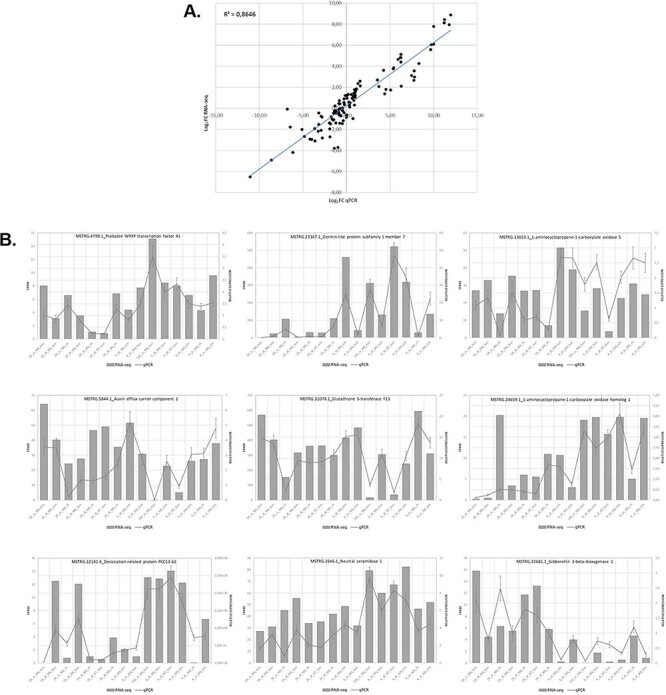
Relationship between levels of expression obtained by RNA-seq and RT-qPCR. (A) Log2 fold change in gene expression determined by plotting RNA-seq data against log2 fold change in gene expression assessed by qPCR. (B) Graphs showing similar trends in the expression levels of individual genes determined by RNA-seq and RT-qPCR analyses.

We assessed the expression of 9 genes selected from 15 samples using RT-qPCR to validate the expression results obtained in the analysis of the RNA-seq data. Overall, the RT-qPCR analysis were similar to the results obtained in the RNA-seq analysis. Figure 5 illustrates the relationship between the RNA-seq and RT-qPCR data, showing the similarity between the data obtained in the two analyses. Figure 5A illustrates the level of correlation between the RNA-seq and RT-qPCR data, which exhibited and *R*^2^ = 0.86, and Figure 5B illustrates the similarity in expression levels of individual genes as determined from the RNA-seq data or the RT-qPCR data.

## Discussion

### Suggested use of the data contained in the OakRootRNADB

The constructed database and web page user interface provide the ability to investigate in-depth the molecular mechanisms regulating postemergence taproot growth and similarities in gene expression and regulatory mechanisms in taproots roots versus lateral. It provides the ability to systematically analyze both CDS and non-CDS and to identify targets of soluble RNAs or miRNA. This information can provide essential knowledge on the molecular network regulating taproot and lateral development, determining the root architecture in pedunculate oak trees. The data available in OakRootRNADB can also be used by root research community to explore how the seedling root system of pedunculate oaks will respond to environmental cues. A comparison of the genes involved in root development in a long-lived, perennial tree species versus an annual plant, such as *Arabidopsis*, can help to determine how life cycle shapes the transcript profiles of roots. Our database provides a broad transcriptomic perspective that will enable biologists the function of specific genes in relation to other parameters (root type, season, etc.). The database will be extremely useful for researchers who want to predict root growth in tree nurseries and root response to water shortage.

### Conclusion

The constructed OakRootRNADB database is provided with a user-friendly and intuitive interface that provides the ability to analyze the RNA-seq data deposited in the database. We illustrate how to use the OakRootRNADB database and how it facilitates the analysis of both CDS and non-CDS involved in the regulation of taproot and lateral root growth. The OakRootRNADB database contains both CDS and non-CDS obtained from an RNA-seq analysis of pedunculate oak and enables one, for the first time, to obtain a broad picture of the genes involved in regulating the growth and development of a long-lived root system comprised of both a taproot and lateral roots. The database can be used as a starting point for research on mRNA and ncRNA associated with pedunculate oak, as well as other perennial plant species, especially trees.

## Supplementary Material

baac097_Supp

## Data Availability

The data discussed in this publication have been deposited in NCBI’s GEO (30) and are accessible through GEO Series accession number GSE181860 (https://www.ncbi.nlm.nih.gov/geo/query/acc.cgi?acc=GSE181860).
