# Supplementary figures and images for: OakRootRNADB—a consolidated RNA-seq database for coding and noncoding RNA in roots of pedunculate oak (Quercus robur)
Source: Database (Oxford). 2022 Nov 17;2022:baac097. doi: 10.1093/database/baac097 (PMC9670740; doi:10.1093/database/baac097)

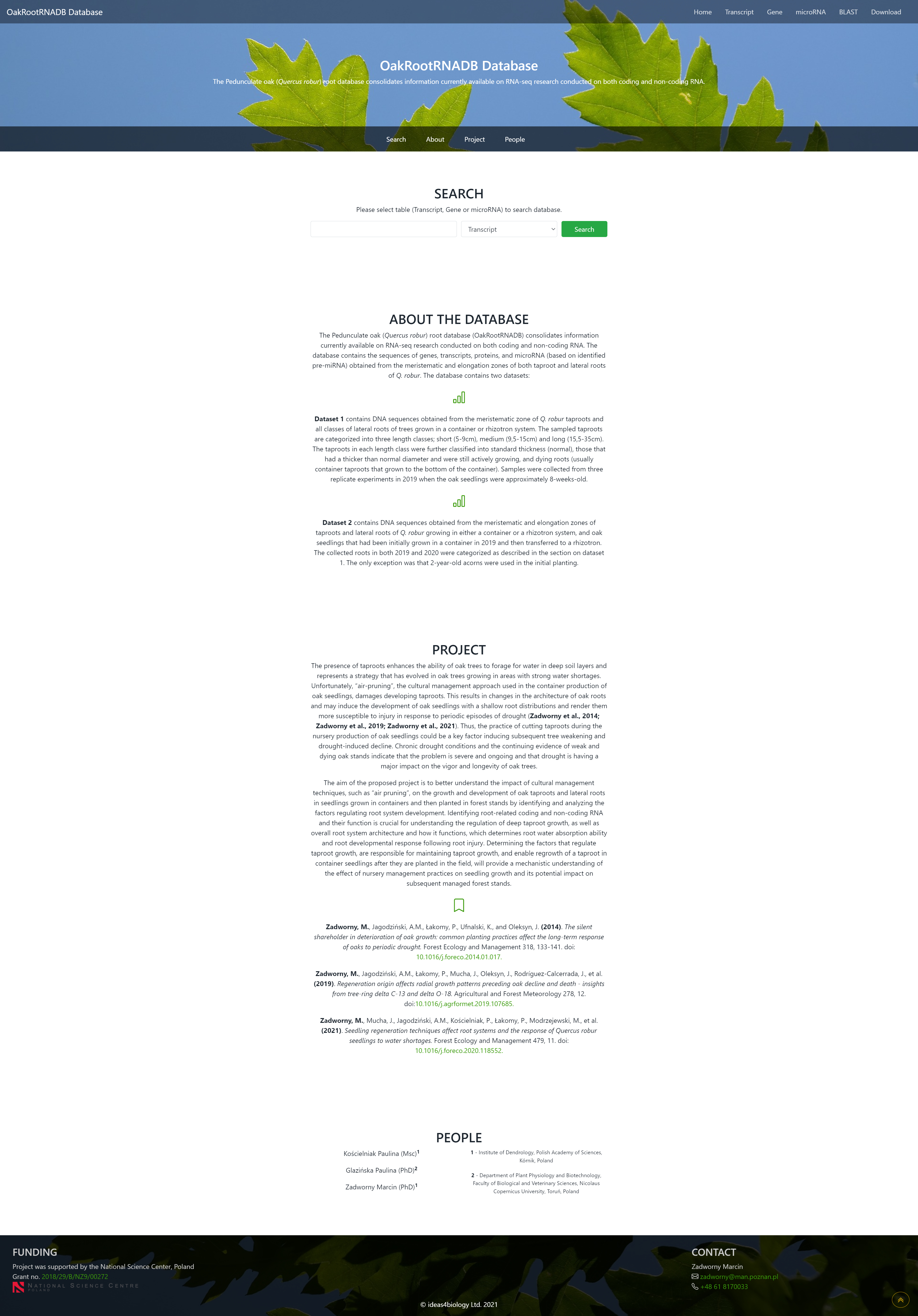

Supplement: baac097_Supp [file baac097_supp.zip › suppl_data/Supplementary Figure 1.jpg]

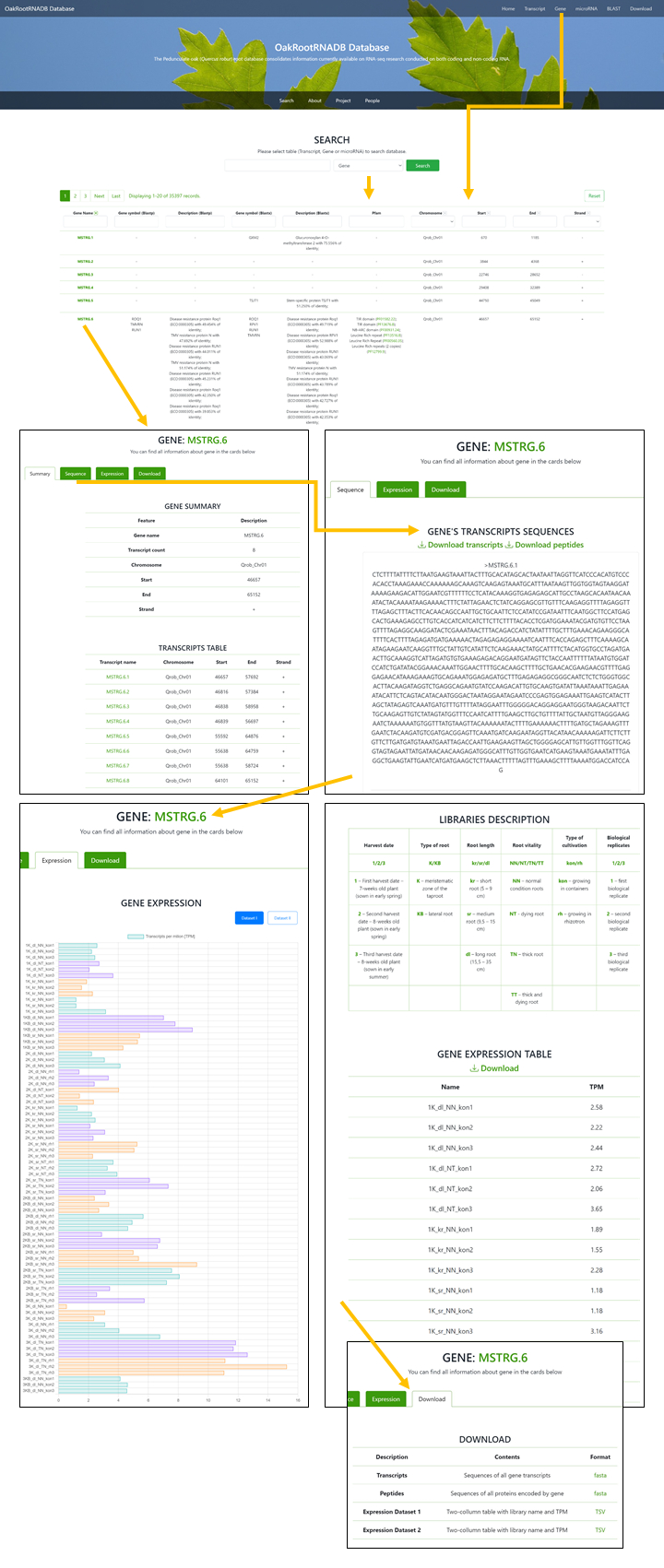

Supplement: baac097_Supp [file baac097_supp.zip › suppl_data/Supplementary Figure 2.jpg]

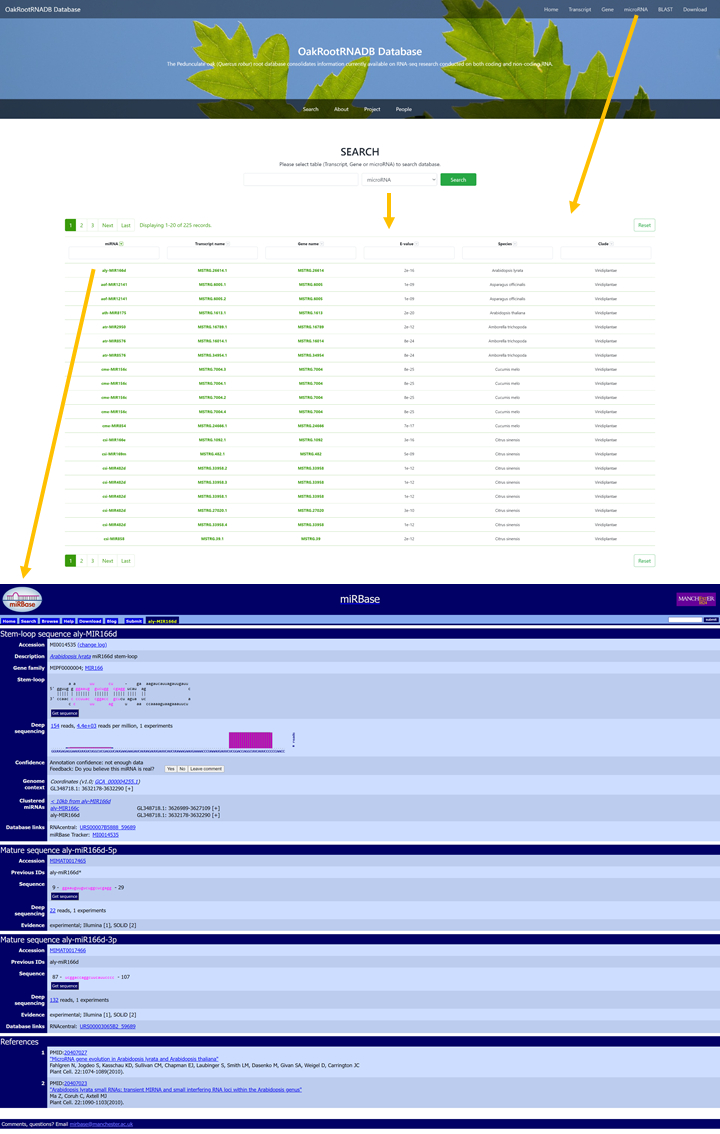

Supplement: baac097_Supp [file baac097_supp.zip › suppl_data/Supplementary Figure 3.jpg]
